# Preferential Autoimmune Response in Prostate Cancer to Cyclin B1 in a Panel of Tumor-Associated Antigens

**DOI:** 10.1155/2014/827827

**Published:** 2014-04-17

**Authors:** Liping Dai, Jitian Li, Rosalia Ortega, Wei Qian, Carlos A. Casiano, Jian-Ying Zhang

**Affiliations:** ^1^Department of Biological Sciences, The University of Texas at El Paso, El Paso, TX 79968, USA; ^2^Center for Health Disparities and Molecular Medicine, Department of Basic Sciences, Loma Linda University School of Medicine, Loma Linda, CA 92354, USA

## Abstract

Previous studies have demonstrated that sera from patients with prostate cancer (PCa) contain autoantibodies that react with tumor-associated antigens (TAAs). Autoantibodies to cyclin B1 and fourteen other TAAs were detected by enzyme-linked immunosorbent assay (ELISA) and Western blotting in 464 sera from patients with PCa, benign prostatic hyperplasia (BPH), and other controls. Autoantibodies to cyclin B1 were detected in 31.0% of sera from randomly selected patients with PCa versus 4.8% in sera with BPH. In the further analysis, 31.4% of sera from PCa patients at the early stage contained anti-cyclin B1 autoantibody, and even 29.4% of patients who had normal prostate-specific antigen (PSA) levels in their serum samples were observed anti-cyclin B1 positive. The cumulative positive rate of autoantibodies against seven selected TAAs (cyclin B1, survivin, p53, DFS70/LEDGFp75, RalA, MDM2, and NPM1) in PCa reached 80.5%, significantly higher than that in normal control sera. In summary, autoantibody to cyclin B1 might be a potential biomarker for the immunodiagnosis of early stage PCa, especially useful in patients with normal PSA level. This study further supports the hypothesis that a customized TAA array can be used for enhancing anti-TAA autoantibody detection, and it may constitute a promising and powerful tool for immunodiagnosis of PCa.

## 1. Introduction

Prostate cancer (PCa) is the most frequently diagnosed cancer in men in the USA and the second leading cause of male cancer deaths after lung cancer, with an estimated 233,000 new cases and 29,480 deaths in 2014 [1]. It also presents a health disparity problem given its disproportionately high incidence and mortality among African American (AA) men [2]. Although effective surgical and radiation treatments exist for clinically localized PCa, metastatic PCa remains essentially incurable. Early diagnosis is the key for treatment and management of PCa. At present, the only widely accepted screening tool for PCa is prostate-specific antigen (PSA). PSA is known to be a sensitive prostate-specific test; however, it is not PCa specific and hence lacks the specificity in differentiating between PCa from other prostate conditions such as prostatitis and benign prostatic hyperplasia (BPH). In addition, obesity lowers PSA levels, which leads to delayed detection of PCa and worse clinical outcome [3]. Of particular concern is the observation that obese AA men with low PSA levels have more aggressive prostate tumors than non-AA men [4]. PSA testing has been the focus of controversy in recent years given its low specificity, which has led to a large number of unnecessary biopsies and diagnostic procedure [5]. Therefore, a critical unmet need in the diagnosis and management of PCa is the development of novel noninvasive markers that can complement PSA testing [6].

The human immune system in cancer appears to sense aberrant tumor-associated antigens (TAAs) as foreign antigens and have the capability to respond to these antigens by producing autoantibodies which are generally called anti-TAA antibodies [7]. In recent years, the potential utility of TAAs and anti-TAA antibody systems as early cancer biomarker tools to monitor therapeutic outcomes or as indicators of disease prognosis has been extensively explored [8].

Cyclin B1 is known to regulate the G2/M transition in the cell cycle. Recent studies have demonstrated aberrant expression of cyclin B1 in several malignant cancers, including breast cancer [9], esophageal squamous cell carcinoma [10], nonsmall cell carcinoma [11], gastric cancer [12], and hepatocellular carcinoma [13]. Higher frequency of anti-cyclin B1 autoantibody was observed in hepatocellular carcinoma and the basis for autoimmune response to cyclin B1 might include aberrations in cyclin B1 regulation leading to altered protein structure or increased expression which results in stimulation of immune reactions [14]. Cyclin B1 has been also implicated in PCa progression as a downstream target of the Notch signaling pathway [15]. Anti-cyclin B1 autoantibodies were recently detected in Caucasian and AA PCa patients, although antibody responsiveness was lower in the AA patients and associated with gene polymorphisms in a particular Fc*γ* receptor allele [16]. It was proposed that since immunity to cyclin B1 might play a protective role, the low anti-cyclin B1 antibody responsiveness in AA patients could be associated with the higher PCa mortality observed in this racial group [16].

This study was initiated to investigate the potential use of the cyclin B1 antigen-antibody system in the detection of PCa. The results demonstrated that autoantibodies to cyclin B1 were detected in 31.0% of PCa sera but only in 4.8% of sera from patients with BPH. In addition, the data has also indicated that the potential usefulness of using antibodies to TAAs miniarray as immune markers for diagnosis of PCa can be enhanced by addition of other selected TAAs.

## 2. Material and Methods

### 2.1. Serum Samples

In the present study, 174 serum samples from patients with PCa were originally collected at Loma Linda University Medical Center. Thirty-five patients with early stage PCa (I/II stage) who had been drawn blood samples several days before surgical operation were obtained from serum bank in the Autoimmune Disease Center at The Scripps Research Institute (La Jolla, CA), in which 55 serial serum samples from 18 PCa patients included at least two to four samples obtained at different time points before and after surgery treatment. The information of PSA in the sera from 35 patients was available. 51.4% (18/35) sera had abnormal PSA level (≥4 ng/mL), whereas 17 (48.6%) had normal level (<4 ng/mL) [17]. In addition, 21 BPH sera, 89 normal human sera (NHS) sera, 51 sera from patients with systemic lupus erythematosus (SLE), and 94 sera from patients with progressive systemic sclerosis (PSS) were also obtained from serum bank in the Cancer Autoimmunity Research Laboratory of The University of Texas at El Paso (UTEP) and used as controls in this study. Normal human sera were collected from adults during annual health examinations in people who had no obvious evidence of malignancy. This study was approved by the Institutional Review Board of UTEP and Collaborating Institutions.

### 2.2. Purification of Recombinant Proteins

In the present study, a total of 15 TAAs including cyclin B1, IMP1, p62, Koc, p53, survivin, p16, CAPER*α*, p90, RalA, NPM1, MDM2, 14-3-3 *ζ*, c-Myc, and DFS70/LEDGFp75 were selected for expression of recombinant proteins. The purification of IMP1, p62, Koc, p53, c-Myc, cyclin B1, survivin, p16, and p90 was described previously [18, 19]. Plasmid pET-CAPER*α* carrying CAPER*α* cDNA was derived from the previous study [20]. cDNA encoding RalA amplified by PCR from a human expressed sequence tag (EST) clone (#BM560822) was subcloned into pET28a vector. NPM1 construct GFP-NPM WT (plasmid ID: 17578), MDM2 construct pGEX-4T MDM2 WT (plasmid ID: 16237), and 14-3-3 *ζ* construct GST-14-3-3 WT (plasmid ID: 1944) purchased from Addgene were subcloned into pET28a vector. A pET-28a plasmid encoding full-length DFS70/LEDGFp75 was purchased from Novagen (Novagen, Madison, WI). Expression of adequate amounts of recombinant protein was examined in SDS-PAGE and Coomassie blue staining was used to determine that expression products of expected molecular sizes were produced. In addition, Western blotting analysis was used to confirm that the bands seen in SDS-PAGE were reactive with reference antibodies.

### 2.3. Enzyme-Linked Immunosorbent Assay (ELISA)

The autoantibodies against 14 TAAs in all of sera were tested by ELISA, which was described in detail previously [21]. The optical density (OD) value of each well was read at 405 nm, and the cut-off value for determining a positive reaction was designated as the mean absorbance of the 89 normal sera plus 3 standard deviations (mean + 3SD). Each sample was tested in duplicate. Each run of ELISA included 8 NHS representing a range of absorbance above and below the mean of 89 normal human sera, and the average OD value of 8 NHS was used to normalize all absorbance values to the standard mean of the entire 89 normal samples.

Cancer sera were also tested against three cellular antigens: SSA52, SSA60, and SSB that are known antigenic targets of autoantibodies in the prototype autoimmune disease, SLE and PSS [22], which could help to determine the extent of specificity of TAAs in cancer.

### 2.4. Western Blotting and Serum Absorption

Serum samples that were determined to contain autoantibodies by ELISA were further tested by immunoblotting to confirm the immunoreactivity of the sera. The purified recombinant proteins were transferred to a nitrocellulose membrane and then incubated with patient sera diluted 1 : 200, and finally incubated with HRP-conjugated goat anti-human IgG diluted 1 : 10000 (Santa Cruz Biotechnology, Inc. Santa Cruz, CA, USA). Positive signals were captured by autoradiography using chemiluminescence (Piece Biotechnology, Rockford, IL), according to the manufacturer's instructions. To confirm specificity of Western blotting, sera at the above dilutions were absorbed with recombinant protein (final concentration: 0.03 *μ*g/mL) overnight at 4°C, the mixture was centrifuged and the supernatant was examined in Western blotting.

### 2.5. Statistical Analysis

*χ* ^2^ tests were used to determine whether the frequency of autoantibodies to TAAs in each cohort of patient sera was significantly higher than that in sera from normal individuals. Two significant levels (0.05 and 0.01) were used. The sensitivity and specificity of the test based on combination of autoantibodies were performed using the receiver operating characteristics (ROC) curve analysis, leading to estimates of the area under curve (AUC), with 95% confidence intervals (CI). Statistical analysis was carried out in SPSS software, version 15.0.

## 3. Results

### 3.1. Frequency of Autoantibody to Cyclin B1 in Prostate Cancer Sera

**Figure 1:**
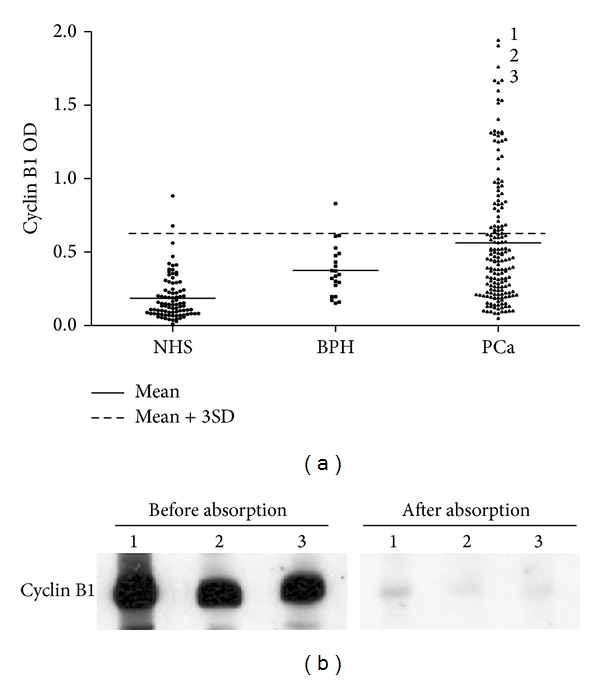
(a) Antibody titers to cyclin B1 in ELISA were expressed as optical density (OD) units. The mean + 3SD of normal human sera are shown in relationship to PCa and BPH. (b) Western blotting analysis shows antibody reactivity to cyclin B1 before and after absorption in nitrocellulose membrane strips blotted with purified recombinant cyclin B1 protein. Lanes 1–3 are sera from PCa patients and the corresponding OD values of these sera are indicated in panel (a). In general, OD values in ELISA were related to intensity of signals in Western blotting and the signals decreased obviously after sera absorption.

As shown in Figure 1(a) and Table 1, 54 of 174 (31.0%) PCa sera had autoantibodies to cyclin B1 with OD values over cut-off higher than NHS (2.2%). Only the OD value in one BPH serum was over the cut-off line. All of sera with OD value over the cut-off in ELISA were confirmed by Western blotting using purified cyclin B1 protein as antigen. Forty-nine of 54 (90.7%) with higher OD value for cyclin B1 from PCa were also positive in Western blotting. In general, higher OD value in ELISA gave the stronger signals in Western blotting, and, after the sera were absorbed using purified cyclin B1 protein, the signals were obviously decreased and they even disappeared (Figure 1(b)). The variations between the two different immunological assays might be related to the increased sensitivity of ELISA over Western blotting, but other possibilities could be related to recognition of different epitopes on the same antigen in the two assays.

### 3.2. Simultaneous Use of Both PSA and Cyclin B1 as Markers in Prostate Cancer Early Detection

In the present study, 35 sera from patients with early stage PCa were available for studies to evaluate the diagnostic value of anti-cyclin B1 in the early detection of PCa and determine the relationship of PSA to anti-cyclin B1 responses (Table 2). 31.4% of 35 patients were observed immunoresponse to cyclin B1. Eighteen of 35 (51.4%) PCa sera had abnormal serum PSA level (>4 ng/mL). Of interest was that 5 of 17 (29.4%) PCa sera with normal PSA level (<4 ng/mL) were anti-cyclin B1 autoantibody positive. If both anti-cyclin B1 and PSA were simultaneously used as diagnostic markers, 23 out of 35 (65.7%) PCa patients could be correctly identified. Elevated PSA and anti-cyclin B1 appear to be independent but supplementary serological markers for the diagnosis of early stage PCa.

### 3.3. Anti-Cyclin B1 Autoantibody Level Was Increased after Surgery

**Figure 2:**
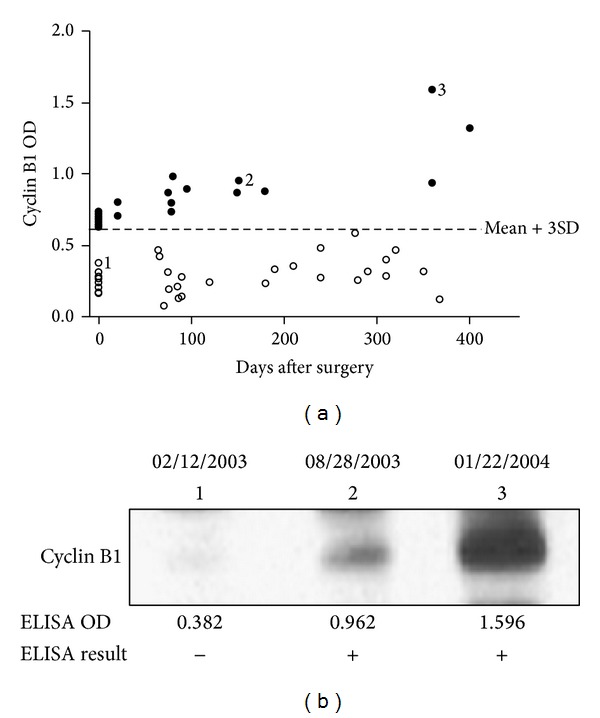
(a) Distribution of cyclin B1 antibody responses in different time points before and after surgery treatment. Anti-cyclin B1 antibody levels (OD values) in the 55 samples from 18 PCa patients were shown. Nineteen serum samples that are positive for anti-cyclin B1 are shown as filled black dots. The other samples are shown as unfilled circles. (b) Western blotting result of serial sera from a representative patient. Lane 1 is the sample before surgery and lanes 2 and 3 are ones after surgery.

Fifty-five serial serum samples from 18 PCa patients were also detected for the expression of autoantibodies against cyclin B1. Serum samples from all these patients had been collected after the diagnosis of PCa and serum samples were also collected every three months after they had been taken surgical resection. At least two to four samples after surgery were available for each patient. These are very valuable serial serum samples from the patients with surgery and the detection of autoantibodies of these samples might reflect the prognosis after surgery. As shown in Figure 2(a), only six serum samples from 18 PCa patients before surgery reacted over the cut-off OD value of cyclin B1 autoantibody. However, 13 blood samples collected after surgery showed positive result in ELISA with higher OD value. There is likely association between cyclin B1 antibody level and the time before diagnosis. The closer to the time of diagnosis, the higher autoantibody level performed. All of the 55 serum samples were also analyzed by Western blotting to confirm the results from ELISA.  Figure 2(b) shows a representative patient, who had no anti-cyclin B1 antibody before surgery (02/12/2003); however, the antibody weakly occurred six months after surgery (08/28/2003) and showed increased reactivity one year after surgery (01/22/2004).

### 3.4. Autoantibody Responses to Other 14 TAAs in Prostate Cancer Sera

In addition to cylcin B1, we have also detected 14 other TAAs, including nine oncoproteins such as three IMP proteins (IMP1, IMP2/p62, and IMP3/Koc) [23], CIP2A/p90 [24], RalA [25], c-Myc [26], survivin [27], 14-3-3 *ζ* [28], and MDM2 [29], four tumor suppressors such as p53 [30], CAPER*α*/HCC1 [20], p16 [31], and NPM1 [32], and the stress survival protein DFS70/LEDGFp75 [33].

**Figure 3:**
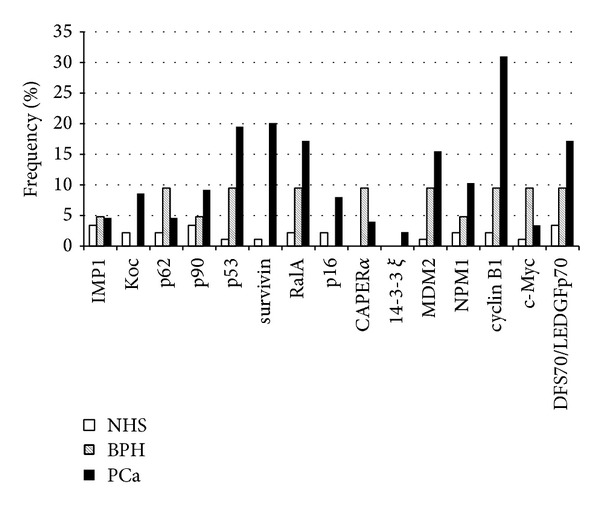
Percentage of autoantibody responses to a panel of 15 TAAs. The percentages refer to antibody titers exceeding the mean + 3SD of normal human sera from ELISA. In this panel of 15 TAAs, the highest reactivity antigen was cyclin B1.

Table 1 and Figure 3 show that PCa also contained autoantibodies to 14 other TAAs in addition to cyclin B1. Table 1 shows the frequency of autoantibodies to the panel of 15 TAAs using ELISA. Higher frequency of antibodies against individual TAA in PCa was found with TAAs such as survivin, p53, RalA, DFS70/LEDGFp75, MDM2, and NPM1 compared to normal human sera (*P* < 0.01) besides cyclin B1. Notably, in BPH, antibody frequency to any individual TAA ranged from 0 to 9.5% and there was no significant difference with NHS. The reactivity of anti-TAAs antibodies in normal human sera was very low, ranging from 0 to 3.4% to any individual TAA. Sera from patients with systemic rheumatic autoimmune disorders (SLE and PSS) also showed very low frequency of 15 anti-TAAs with no more than 6% and established that antibody reactivities observed were cancer specific and not attributable to nonspecific immunoreactivity.

It was important to establish that autoantibodies to TAAs were not the result of some unusual reactivity to intracellular antigens. All 174 PCa sera were analyzed for antibodies to the autoantigens SSA52, SSA60, and SSB which are commonly used biomarkers in systemic rheumatic autoimmune diseases, and none of the cancer sera showed reactions above the cut-off OD, whereas SLE and PSS sera showed the expected percentage of positive reactors (data not shown).

### 3.5. Combination of Autoantibodies to 7 TAAs in the Diagnosis of Prostate Cancer

**Figure 4:**
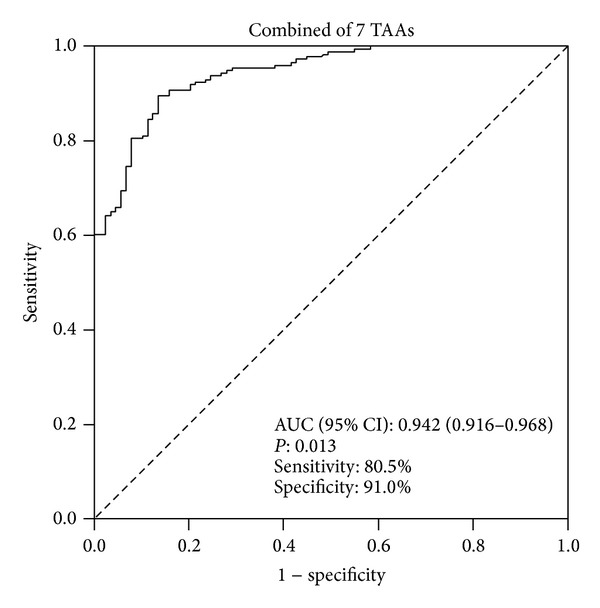
Sensitivity and specificity of ELISA based on combined seven TAAs (cyclin B1, survivin, p53, RalA, DFS70/LEDGFp75, MDM2, and NPM1) for differentiating between PCa (*n* = 174) and normal controls (*n* = 89).

It has been demonstrated that although no single TAA was uniquely associated with a particular type of cancer when panels or mini-arrays of TAAs were used in antigen platforms, some types of cancer were more reactive to certain specific TAAs. In this study, cyclin B1 appeared to be the most reactive antigen in prostate cancer and an analysis was performed to determine whether a subset and not necessarily all the 15 TAAs (Table 1) was sufficient to achieve optimal antibody frequency. Besides cyclin B1, there were six other autoantibodies that showed significant higher frequency in PCa sera than in NHS, such as survivin, p53, RalA, DFS70/LEDGFp75, MDM2, and NPM1. Subsequently, we used ROC curve to analyze the diagnostic value of combination of these 7 autoantibodies in PCa compared to normal individuals, which showed that the sensitivity reached 80.5% and the specificity 91% with AUC at 0.942 (95% CI: 0.916–0.968) (Figure 4).

## 4. Discussion

The current study shows that autoantibodies to cyclin B1 were detected in 31.0% of PCa sera, which is significantly different from BPH (4.8%) and other disease controls such as SLE and PSS, as well as normal individuals. The different factors leading to the increased production of such autoantibodies are not completely understood but many investigators have been interested in the use of autoantibodies as serological markers for cancer diagnosis, especially because of the general absence of these antibodies in normal individuals and in noncancer conditions [21, 34]. Our results suggested that cyclin B1 autoantibody might be taken as a potential immunodiagnostic biomarker in PCa as part of a TAA array for autoantibody detection.

Although PSA serum levels are the most commonly used diagnostic test for PCa to date, its specificity is less than 50%, resulting in frequent false-positive results [35]. Many studies have proposed the use of panels of markers including PSA to increase the specificity and sensitivity of diagnosis but this is still far from routine use. Xie and colleagues [36] designed a new multiplex assay termed “autoantibody + PSA” assay to develop a reliable platform that will enable the diagnosis of patients with prostate cancer relative to nonmalignant cases. They found that PSA alone had a sensitivity of 52% and specificity of 79% in PCa patients, whereas the autoantibody + PSA platforms showed a sensitivity of 79% and a specificity of 84% in PCa patients. In the present study, detecting 35 patients at early stage of PCa shows that 31.4% sera were observed anti-cyclin B1 autoantibody positive. If both anti-cyclin B1 and PSA were simultaneously detected, the percentage of patients with early stage PCa who could be correctly identified reached 65.7%.

In recent years, the potential utility of TAAs and anti-TAA antibody systems as early cancer biomarker tools to monitor therapeutic outcomes, or as indicators of disease prognosis, has been extensively explored [8, 37, 38]. It has been reported that cyclin B1 was associated with recurrence in hepatocellular carcinoma [39], poor prognosis in gastric cancer [12], and poor survival in breast cancer [40]. Of interest in the present study was that six out of eighteen PCa patients showed the increased anti-cyclin B1 antibody after surgery. It is hypothesized that in a patient where a certain anti-TAA antibody is detected, change in antibody levels might reflect change in tumor status or tumor burden related to therapy [41]. Although it was uncertain to determine whether there was any relation between anti-cyclin B1 and clinical outcome after radical prostatectomy surgery; because of the lack of further follow-up information on patients after surgery, anti-cyclin B1 autoantibody still might be a potential biomarker for the prognosis of PCa after surgery treatment.

PCa recurrence after prostatectomy surgery often gives rise to more aggressive, metastatic tumors, especially among African American men [42]. Given that autoantibodies in PCa patients may be sensors or sentinels of events associated with prostate tumorigenesis [43], it would be tempting to speculate that the rise in cyclin B1 autoantibodies may reflect changes in the expression or activity of this protein in the patients. Whether these changes are associated with tumor recurrence, particularly in African American men, whose immunity to cyclin B1 might be associated with increased tumor aggressiveness [16], remains to be determined. Thus, our novel preliminary finding indicating increased anti-cyclin B1 antibody after surgery is encouraging and warrants future follow-up studies. Prospective studies on cohorts of racially and ethnically diverse patients with PCa, prior to and after radical prostatectomy, may reveal the potential of a cyclin B1 antibody-based assay to predict PCa recurrence after surgery. In addition, comprehensive studies on the biology of cyclin B1 in prostate cancer progression and metastatic castration-resistant PCa are guaranteed to better understand the significance of these autoantibodies.

The use of autoantibodies as serological markers for cancer diagnosis has attracted much attention in recent years [21, 37, 38, 44, 45]; however, enthusiasm for this approach has been tempered by low sensitivity when individual antigen-antibody reactions were studied [46]. We have observed that this drawback can be overcome by using a panel of carefully selected TAAs to achieve the sensitivity and specificity required to make immunodiagnosis a feasible adjunct to tumor diagnosis [18, 21, 47, 48]. One of our previous studies showed that detection of autoantibodies in cancer can be enhanced by using a mini-array of seven TAAs as target antigens which included c-Myc, p53, cyclin B1, IMP2/p62, Koc, IMP1, and survivin [21, 46]. Antibody frequency to any individual TAA ranged from 10.8% to 24.6% in HCC. With the addition of TAAs to a final total of seven antigens, there was a stepwise increase of positive antibody reactions up to 56.9% in HCC.

In the current study, the TAA panel for detecting anti-TAAs antibodies in PCa has been improved by a mini-array of seven TAAs including cyclin B1, survivin, p53, RalA, DFS70/LEDGFp75, MDM2, and NPM1. Almost eighty percent of PCa could be detected by using this seven TAAs panel.

Important future studies with larger sample size should be performed to determine whether anti-cyclin B1 autoantibody might be useful in identifying early PCa in high-risk individuals and whether it could be used as supplementary serological marker for the early diagnosis of PCa, in combination with PSA and digital rectal examination, or for disease recurrence after treatment with surgery, hormonal therapy, or chemotherapy. Furthermore, additional efforts should be aimed at increasing both the sensitivity and specificity of serum autoantibodies as PCa markers by expanding TAA arrays to include antigens that might be more selectively associated with PCa and not with other cancers.

## Figures and Tables

**Table 1:** Frequency of autoantibodies against 15 TAAs in prostate cancer and BPH.

Conditions	*N*	cyclin B1	IMP1	Koc	p62	p90	p53	survivin	RalA	p16	CAPER*α*	14-3-3 *ζ*	MDM2	NPM1	c-Myc	DFS70
PCa	174	54 (31.0)^∆^	8 (4.6)	15 (8.6)	8 (4.6)	16 (9.2)	34 (19.5)^∆^	35 (20.1)^∆^	30 (17.2)^∆^	14 (8.0)	7 (4.0)	4 (2.3)	27 (15.5)^∆^	18 (10.3)*	6 (3.4)	30 (17.2)^∆^

NHS	89	2 (2.2)	3 (3.4)	2 (2.2)	2 (2.2)	3 (3.4)	1 (1.1)	1 (1.1)	2 (2.2)	2 (2.2)	0 (0.0)	0 (0.0)	1 (1.1)	2 (2.2)	1 (1.1)	3 (3.4)

BPH	21	1 (4.8)	1 (4.8)	0 (0.0)	2 (9.5)	1 (4.8)	2 (9.5)	0 (0.0)	2 (9.5)	0 (0.0)	2 (9.5)	0 (0.0)	2 (9.5)	1 (4.8)	2 (9.5)	2 (9.5)

SLE	51	3 (5.9)	2 (3.9)	2 (3.9)	2 (3.9)	3 (5.9)	0 (0.0)	2 (3.9)	2 (3.9)	2 (3.9)	2 (3.9)	1 (2.0)	2 (3.9)	3 (5.9)	2 (3.9)	1 (2.0)

PSS	94	3 (3.2)	1 (1.1)	0 (0.0)	0 (0.0)	2 (2.1)	3 (3.2)	4 (4.3)	2 (2.1)	2 (2.1)	0 (0.0)	3 (3.2)	3 (3.2)	2 (2.1)	0 (0.0)	2 (2.1)

PCa: prostate cancer, NHS: normal human serum, BPH: benign prostatic hyperplasia, SLE: systemic lupus erythematosus, and PSS: progressive systemic sclerosis.

^∆^ *P* < 0.01 compared to NHS.

*P < 0.05 compared to NHS.

**Table 2:** Sensitivity of combined use of both PSA and cyclin B1 antibody in early prostate cancer detection.

PSA level (ng/mL)	Cyclin B1 antibody		Total
	+	−	
≥4	6 (*A*)	12 (*B*)	18
<4	5 (*C*)	12 (*D*)	17

Total	11	24	35

Sensitivity (%) of PSA is *A* + *B*/(*A* + *B* + *C* + *D*) = 18/35 = 51.4%.

Sensitivity (%) of cyclin B1 is (*A* + *C*)/(*A* + *B* + *C* + *D*) = 11/35 = 31.4%.

Sensitivity (%) of both PSA and cyclin B1 combined is (*A* + *B* + *C*)/(*A* + *B* + *C* + *D*) = 23/35 = 65.7%.

Cyclin B1 positive in individuals with PSA <4 ng/mL is *C*/(*C* + *D*) = 5/17 = 29.4%.

## Abbreviations

AA: African American
BPH: Benign prostatic hyperplasia
ELISA: Enzyme-linked immunosorbent assay
GST: Glutathione S transferase
HRP: Horseradish peroxidase
NHS: Normal human sera
OD: Optical density
PCa: Prostate cancer
PCR: Polymerase chain reaction
PSA: Prostate-specific antigen
PSS: Primary sjögren's syndrome
SLE: Systemic lupus erythematosus
TAAs: Tumor-associated antigens.
